# 4.B. Workshop: Addressing backlogs and managing waiting lists during and beyond the COVID-19 pandemic

**DOI:** 10.1093/eurpub/ckac129.209

**Published:** 2022-10-25

**Authors:** Organised by: European Observatory on Health Systems and Policies, Chair persons: Ewout Van Ginneken (Germany), Sarah Reed (UK)

## Abstract

As COVID-19 cases started to rise in early 2020 and hospitalisation rates increased, health systems began to postpone non-emergency (elective) procedures to keep capacity available for COVID-19 patients, and to avoid elective patients being infected. This has subsequently led to longer waiting lists and waiting times in virtually all countries. Issues around staff recruitment and retention, which have been exacerbated by the COVID-19 pandemic, have further aggravated the problem. For patients with common elective surgeries, such as hip and knee replacements, the backlog in care means that improvements in health and quality of life are postponed. For urgent care, such as missed chemotherapy sessions for cancer care, the delays can have more severe consequences. For other patients, the postponement of specialist appointments may lead to missed referrals for serious ailments. Increasingly also primary care has become affected leading to late diagnosis of chronic diseases, as well as inadequate follow up and control of these patients. Each delay in diagnosis and treatment may worsen health problems, prolong recovery and decrease the patients’ chances of survival. Countries are now left playing catch-up on these backlogs. There is however great uncertainty regarding the size of the backlogs, how much current and future capacity will be required to address them, and how much provider and workforce capacity will be needed for COVID-19 patients which will detract capacity for non-COVID patients. If health systems do not manage to reduce the backlog, they risk worsening health outcomes and wasting important health gains made in the last years. This workshop will discuss what we know about (1) the level of service disruptions and resulting backlog, (2) the drivers of backlog, and (3) which policies countries are using to address this. The workshop will conclude with an audience discussion about how to measure the true size of the backlog, the policy options for overcoming backlog and key priorities for further research.

**Key messages:**

• The COVID-19 pandemic has led to substantial disruptions in care delivery leading to care backlogs in virtually all countries.

• Countries have various policy options to tackle backlogs and bring down waiting times in the wake of the pandemic.

